# Association of serum sodium levels and diabetes: A critical analysis

**DOI:** 10.1111/1753-0407.13366

**Published:** 2023-02-03

**Authors:** Usmaan Al‐Shehab, Hasan Zia, Brandon Goodwin, David F. Lo

**Affiliations:** ^1^ Department of Medicine Rowan University School of Osteopathic Medicine Stratford New Jersey USA; ^2^ American Preventive Screening & Education Association (APSEA) Stratford New Jersey USA; ^3^ Department of Biology, Rutgers The State University of New Jersey New Brunswick New Jersey USA

We read with pleasure the article by Cheng et al,^1^ titled “Serum sodium level is inversely associated with new‐onset diabetes in hypertensive patients” and would like to offer additional commentary on the extrapolated conclusions regarding the diagnostic criteria for hypertension and diabetes. We hope these perspectives may provide insight into areas that may require further research and improvement.

Cheng et al summarized that serum sodium is an essential electrolytic marker of cardiovascular and endocrine health. Although hypertension and diabetes are the most prevalent diseases globally, there is little research on the association between serum sodium levels and the onset of diabetes. The authors investigated the effect of sodium levels on hypertensive patients (who were not diagnosed with diabetes at the time) from Dongguan City, Guangdong, China. At a median 35.1‐month follow‐up, logistic regression analysis and cubic spline analysis were performed to investigate the association between serum sodium levels and new‐onset diabetes. Logistic regression analysis revealed that an increased serum sodium level was associated with a decreased risk of new‐onset diabetes, and cubic spline analysis revealed that there was a negative linear correlation between serum sodium levels and new‐onset diabetes. However, it may be possible that the diagnostic criteria for hypertension and diabetes may lead to a misdiagnosis in some participants, undermining the results of the study.

First, it is important to consider the effects of errors or biases that can occur in self‐reporting hypertension. The effect of self‐reporting bias is well documented in the literature and can have significant effects on the patient population of the study.^1^ In the case of Cheng et al, it is possible that patients who met the inclusion criteria may not have been included due to self‐reporting of not having hypertension. Conversely, it is possible that patients who did not meet the inclusion criteria self‐reported having hypertension. Additionally, patients may misreport hypertension if they are not aware of their blood pressure level.^2^

Second, it is also possible that patients may have falsely met the inclusion criteria for the study because they take antihypertensive medications. To be particular, patients may be taking antihypertensive medications prophylactically despite not having a diagnosis of hypertension, such as in the case of nifedipine being used to treat Raynaud's phenomenon.^3^ This, therefore, opens the opportunity for patients with low serum sodium levels and no diagnosis of hypertension to meet the inclusion criteria for the study. These two concerns can easily be alleviated by including blood pressure screening for every participant in the study and limiting the diagnostic criteria for hypertension to a systolic blood pressure ≥140 mm Hg and/or a diastolic blood pressure ≥90 mm Hg three times on different days. This would result in a decrease in the number of patients who met the inclusion criteria and strengthen the accuracy of the conclusions.

Third, similar to the concerns addressed previously, the diagnostic criteria of diabetes by the use of hypoglycemic therapy during the follow‐up period may lead to a false diagnosis of diabetes. It is important to consider that hypoglycemic therapy may be prescribed to treat conditions other than diabetes, such as metformin with polycystic ovarian syndrome.^4^ This can be easily corrected by measuring the fasting blood glucose during the follow‐up period to make a definitive diagnosis of diabetes.

Overall, the study relied on self‐reported data on hypertension diagnosis, which is subject to self‐reporting bias. Additionally, the diagnostic criteria for hypertension and diabetes based on medication use may lead to miscategorizing participants in the logistic regression and cubic spline analysis. In the end, we applaud the authors for conducting an interesting and unique study into the associations between serum sodium levels and diabetes. We look forward to reading about future studies that provide insight into the association between hypertension and diabetes.

## FUNDING INFORMATION

No funding was received for this study/paper.

## CONFLICT OF INTEREST STATEMENT

The authors declare that they have no competing interests.
